# New *Vibrio cholerae *O1 Biotype ElTor bacteriophages

**DOI:** 10.1186/1743-422X-2-28

**Published:** 2005-04-11

**Authors:** Anindito Sen, Amar N Ghosh

**Affiliations:** 1 Division of Electron Microscopy, National Institute of Cholera and Enteric Diseases, P-33, C.I.T. Road, Scheme- XM, Beleghata, Kolkata- 700010. India; 2 (Present Address) Laboratory of Structural Biology, Room 1504, Building 50, NIAMS/NIH Bethesda, MD, 20852, USA

**Keywords:** Vibrio, phage, Electron microscopy

## Abstract

We report the presence of three new O1 ElTor vibriophages named AS1, AS2 and AS3, isolated from the sewage and pond waters of the outskirts of Kolkata. A few phages, named AS4, with hexagonal heads and abnormally long tails with typical curly projections were also found in the water samples.

*Vibrio cholerae*, the causative agent of cholera in humans, is classified into two serotypes: O1 and nonO1 [1]. The O1 strains are divided into two biotypes: Classical and ElTor. Before 1961 most epidemics had been caused by the classical biotype. But with the passage of time the classical biotype disappeared from the scenario and the ElTor emerged as the major biotype causing the *Vibrio cholerae *in humans. In 1993, *Vibrio cholerae *serogroup O139 made an explosive appearance and caused a severe epidemic in the Indian continent [2]. The disease cholera spreads rapidly to far off places from the epicenter of its emergence. From the epidemiological point of view it is important to track down the spread of the disease. Phage typing is a widely accepted method for tracking down cholera epidemic [3]. The international phage-typing scheme of Basu and Mukerjee [3] includes five phages (I, II, III, IV and V). But in course of time this typing scheme proved inadequate as a large number of *Vibrio cholerae *strains were found to be untypeable using this scheme. In order to overcome this problem a new typing scheme for ElTor strains was proposed in 1993 [4]. In the recent times vibriophages are found to occur in amazingly in large numbers in the environment around the globe [5-8] which, prompted us to search for new cholera phages from the environmental resources.

Sewage and pond water were collected from different places from the outskirts of Kolkata. During the study period the recorded temperature was about 32–38°C and pH ranged from 7.8 to 10. The sample waters were processed for phage isolation as described previously [9] using *Vibrio cholerae *O1 ElTor (MAK 757) as the propagating strain. The procedure was repeated on nutrient agar or the appearance of plaques. The phages were purified from a single discrete plaque by the soft agar (0.9 %) overlay method. Phage lysates were prepared in nutrient broth. A few drops of chloroform were added to the freshly prepared phage lysate to remove bacterial content in it. The phage lysates (nearly 10^8 ^– 10^9^phages/ml) was subjected to ultracentrifugation at 35,000 r.p.m. for 90 minutes in a Sorval T 865 rotor and phage pellets were obtained. The phage pellets were resuspended in 1 ml of 50 mM; Tris-HCl pH 7.5, 20 mM, MgCl_2 _(TM buffer) to concentrate and the phage was stored at 4°C. The phages was purified on a sucrose step gradient of 10% to 40% as described previously [6] using a Sorval TW 668 swing-out rotor at revolution speed of 35,000 r.p.m. for 75 minutes. The purified phage pellets were resuspended in 1 ml of TM buffer and stored at 4°C. Five microliters of the purified suspensions were deposited directly on Pioloform coated 300-mesh Nickel grids, stabilized with a thin layer of carbon, allowed to adsorb for two minutes and the excess liquid was blotted out. The samples were stained with 2% aqueous uranyl acetate (pH 4.5). Grids were examined in FEI Tecnai 12 Biotwin transmission electron microscope. Measurements of the dimensions of the head (distances between the opposite apices), length and thickness of the tail were done using 'analySIS' software (SIS GmbH, Germany). Calibration was done using catalase crystal with alternate lattice plane spacing of 8.75 nm and 6.85 nm (Agar Scientific Ltd. England). Several enteropathogens were included to test the susceptibility against these phages. Cultures of these enteropathogens were grown to their mid log phases and were plated as lawn on (0.5% NaCl) nutrient agar plate. The lawns are spotted with about 5–7 μl of the lysates. Table 1 shows the result of the phage sensitivity to the different pathogens. It is seen that phages are only sensitive to *Vibrio cholerae *O1 and are resistant to rest of the species.

**Table 1:** Table showing the sensitivity of the newly isolated phages to the different species of enteropathogens

Species	Phage AS1	Phage AS2	Phage AS3
*Vibrio cholerae *O1	Sensitive	Sensitive	Sensitive
*Vibrio cholerae *O139	Resistant	Resistant	Resistant
*Vibrio cholerae *non-O1	Resistant	Resistant	Resistant
non-O139			
*Vibrio parahaemolyticus*	Resistant	Resistant	Resistant
*Escherichia coli*	Resistant	Resistant	Resistant
*Salmonella enterica*	Resistant	Resistant	Resistant
serovar Typhimurium			

We have not included the phage AS4 for the sensitivity study as their number is very small which, may give erroneous results.

Three different types of phages, named AS1, AS2 and AS3 were found. All the three phages have hexagonal heads with long tail (figure 1). Phages AS1 and AS3 have hexagonal heads and contractile tails and falls in the family of *Myoviridae *while phage AS2 has a hexagonal head with non-contractile tail and falls within the family *Siphoviridae *(according to **I**nternational **C**ommittee for the **T**axonomy of **V**iruses; 1982). The dimensions of the head (distances between the opposite apices), length and thickness of the tail of these phages are summarized in table 1. Morphological comparisons of these three phages were made with several other important typing vibriophages that possess hexagonal heads and long tails have been made and are given in table 1.

**Figure 1:**
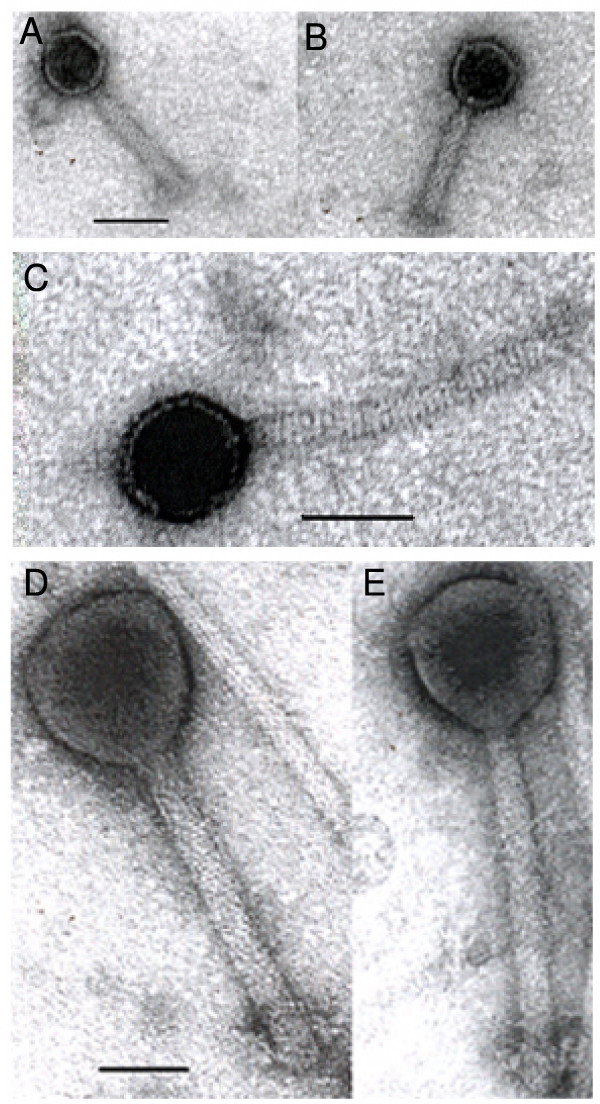
*Vibrio cholerae *O1 Biotype ElTor bacteriophages AS1-3. Panels A and B show the vibriophage AS1. They are contractile in nature and possess similar pattern as seen in the tail of another O1 ElTor typing vibriophage D10 (Chakrabarti et al., (1993). Panels A and B are shown at the same magnification. Panel C show the vibriophage AS2. The tails are non-contractile in nature. Panels D and E show the vibriophage AS3. Panels D and E are shown at the same magnification. The bars in Panels A, C and D: 50 nm.

From table 1 we find that phages AS1, AS2 and AS3 are morphologically different from the other typing vibriophages. While studying these phages we came across few phages (extremely small in number), named AS4, that have the head diameter of nearly equal to 65.24 ± 3.1 nm, straight-tail length nearly 460.20 ± 11.2 nm long and typical curly projection of length 230.20 ± 12.4 nm attached to the free end of the tail. In fact each of the curly projection has a constant contour length of 38.8 ± 5.72 nm. The thickness of the tails is about 10.52 ± 0.86 nm (figure 2). To best of our knowledge, till date, no vibriophage with such abnormally long tails are reported. However, Ackermann and DuBow [10] reported two non-cultivated rumen bacteriophages with such long tails but they do not have any curly projections as seen in AS4.

**Figure 2:**
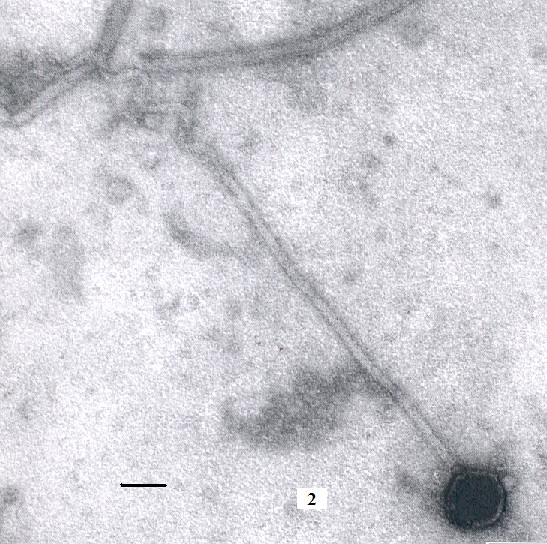
*Vibrio cholerae *O1 Biotype ElTor bacteriophage AS4. The tails are enormously long and non contractile in nature. Typical curly projections are seen at the end of the tails. The number of such phages is extremely rare in the water samples. Bar: 50 nm.

Isolation of these new cholera phages from the sewage and pond waters collected from the outskirts of Kolkata, a high cholera-endemic region, (where a cholera outbreak took place nearly two years back) carries additional significance. Detailed physiochemical studies like host specificity, immunological analysis, study of structural proteins, thermal and light inactivation, growth characteristics and extensive study of their genomes of these newly isolated vibriophages will prove helpful in modifying the present phage typing scheme of *Vibrio cholerae *O1 biotype ElTor untypeable strains in future as it was needed for the old Basu and Mukerjee [3] O1 biotype ElTor typing scheme almost a decade back.

**Table 2:** Morphology of different Vibriophages

Phage	Host	Diameter of head (nm)	Length of tail (nm)	Thickness of tail (nm)	Nature of tail	Reference
**AS1**	*VC *O1 ElTor MAK 757	43.60 ± 2.34	85.21 ± 3.80	13.54 ± 0.91	Contractile tail	Present study
**AS2**	*VC *O1 ElTor MAK 757	44.93 ± 1.35	123.88 ± 5.21	8.83 ± 0.43	Non-contractile tail)	Present study
**AS3**	*VC *O1 ElTor MAK 757	90.1 ± 2.21	193.5 ± 14.5	22.8 ± 1.25	Contractile tail	Present study
**M4 **(O1 ElTor typing phage)	*VC *O1 ElTor MAK 757	97.7 ± 0.03	109.6 ± 0.2	18.2 ± 0.4	Contractile tail	Chattopadhyay et al., 1993
**D10 **(O1 ElTor typing phage)	*VC *O1 ElTor MAK 757	62.9 ± 0.06	101.4 ± 0.3	15.8 ± 0.4	Contractile tail	Chattopadhyay et al., 1993
**MAD-5 **O139 typing phage	*VC *O139 NPR-4	58.0 ± 2.7	141.2 ± 4.8	8.33 ± 0.4	Contractile tail	Chakrabarti et al., 2000
**VE-2 **O139 typing phage	*VC *O139 NPR-4	112.5 ± 1.8	204.0 ± 2.8	23.0 ± 0.2	Contractile tail	Chakrabarti et al., 2000
**Group II **classical typing phage	*VC *Classical 154	62.1 ± 3.1 & 65.5 ± 3.7 nm for the widest & narrowest sections	81.0 ± 3.2	16.6 ± 2.0	Contractile tail	Chatterjee and Maiti, 1984
**Group IV **classical typing phage	VC Classical 154	73.8 ± 3.3 & 83.6 ± 4.0 for the widest & narrowest sections	152.8 ± 8.2	10.7 ± 1.4	Non-contractile tail	(Chatterjee and Maiti, 1984)
**DR1**	VC O26	77.5 ± 0.3	100.0 ± 0.6	19.0 ± 0.4	Contractile tail	(Sarkar et al., 2004) [11]
**DR2**	VC O39	83.3 ± 0.3	111.0 ± 0.8	17.0 ± 0.5	Contractile tail	(Sarkar et al., 2004)
**ΦP15**	VC O1 ElTor Inaba	52.9 ± 9.0 & 40.5 ± 9.5 for the widest & narrowest sections	105.4 ± 3.2	22.5 nm	Contractile tail	(Talledo et al., 2003)

(*VC *stands for *Vibrio cholerae*)
